# Assessment of the Magnitude of Contextual and Individual Demographic Effects on Diabetes Mellitus and Glucose Intolerance in Rural Southwest China: A Multilevel Analysis

**DOI:** 10.1371/journal.pone.0068553

**Published:** 2013-07-16

**Authors:** Ke-wei Wang, Zhan-kun Shu, Le Cai, Jun-Qing Wu, Wei Wei

**Affiliations:** 1 School of Public Health, Fudan University, Shanghai, China; 2 Department of Epidemiology and Social Science on RH, Shanghai Institute of Planned Parenthood Research/WHO Collaborating Centre for Research in Human Reproduction Unit of Epidemiology, Shanghai, China; 3 Luo Ping County Hospital, Yunan Province, Yunan, China; 4 School of Public Health, Kunming Medical University, Kunming, China; College of Pharmacy, University of Florida, United States of America

## Abstract

**Objective:**

This study aimed to determine the contribution of individual and contextual socioeconomic status (SES) to the prevalence of diabetes mellitus and glucose intolerance in the adult population in rural southwest China.

**Methods:**

A population-based cross-sectional study of diabetes was performed in 4801(2152 men) Chinese adults (≥25 years old). Multilevel logistic regression model was used to examine the association between individuals’ and townships’ variables and the prevalence of diabetes mellitus and glucose intolerance.

**Results:**

The age-and gender-standardized prevalence of diabetes mellitus and glucose intolerance were 7.1% (3.6% for undiagnosed) and 8.8% in adults aged ≥25 years, respectively, and increasing with age. Females were more likely to develop diabetes than males. The probability of developing diabetes increased with BMI. Both contextual and individual educational level and yearly household income were found to be negatively associated with the prevalence of diabetes. Residence in communities with a higher percentage of ethnic minorities was associated with higher prevalence of diabetes. Smoking had a protective effect for diabetes, drinking had a positive association with diabetes mellitus and glucose intolerance.

**Conclusions:**

Diabetes mellitus and glucose intolerance are common in rural adults of southwest China by international standards. These results indicate that diabetes mellitus has become a major public health problem in rural areas in southwest China, and strategies aimed at the prevention and treatment of diabetes mellitus and glucose intolerance are needed.

## Introduction

In the past two decades there has been an overall increase of diabetes mellitus prevalence in the developing countries [1], [2], [3]. According to the recent global estimates of the World Health Organization (WHO), the prevalence of diabetes mellitus world-wide is estimated to be 5.4% by 2025 [4], [5], the total number of people with diabetes mellitus is projected to rise from 171 million in 2000 to 366 million in 2030 [3]. A study in China found that there were 92.4 million persons with diabetes mellitus and 148.2 million persons with glucose intolerance among adults 20 years of age or older. Approximately 43.1 million persons with diabetes mellitus and 83.5 million persons with glucose intolerance lived in rural areas [3]. However, because of the methodologies used in these studies, estimates of diabetes mellitus prevalence in the ethnic minority areas were rarely made and are therefore lacking for China, especially for rural areas.

Some studies showed that the aging of the population, obesity, urbanization, physical inactivity and an unhealthful diet have probably contributed to the rapid increase in the diabetes burden in the Chinese population [1], [2], [3], [4], [5]. However, few have studies examined the relative contributions of contextual risk factors to the increased prevalence of diabetes mellitus in the Chinese population, especially among rural populations. Most recent estimates of diabetes mellitus prevalence in China have relied on self-reported data, but since Type 2 diabetes can be asymptomatic for many years before it is diagnosed in a clinical situation, reliance on self-reported information invariably contributes to an underestimation of the true prevalence. Furthermore, such studies failed to provide information on contextual socioeconomic determinants, which are able to substantially increase the risk of future diabetes mellitus.

Increased prevalence of diabetes mellitus and glucose intolerance has also been reported in subjects with low SES [6], [7], [8], [9]. Similarly among Chinese, increased personal income, especially in people with low educational attainment, was identified as a risk factor for diabetes mellitus [10]. Though some studies have suggested that local socioeconomic disparities are related to diabetes prevalence [11], [12], those results may be partly explained by the inverse relationship between diabetes mellitus risk and socioeconomic status (SES) at the individual level [13], [14]. The extent to which local variations in diabetes mellitus prevalence can be attributed to socioeconomic characteristics of the environment itself, independent of person-level characteristics, has been far less studied, and remains a subject of debate. We therefore undertook this study to assess the prevalence of diabetes mellitus and glucose intolerance among the rural population and to examine potential relationships of diabetes mellitus with individual and contextual socioeconomic status (SES).

Located in south-western China, Yunnan, which is one of China's relatively undeveloped provinces, has a total of 16 prefectures, and its population includes 25 ethnic groups with an estimated total population of 45.7 million (2009). About 38% of the province's population are members of minorities, most of who live in mountainous areas. Over the preceding 20 years, the annual gross domestic product has increased from 36.31 billion Chinese Yuan (US$ 9.64 billion) to 616.9 billion Yuan (US$ 90.3 billion). Due to the rapid economic development, significant changes have occurred in the lifestyles of residents of rural and mountainous areas. Among these communities there is a wide variability in level of socioeconomic development and ethnic composition. The risk for diabetes mellitus and glucose intolerance among the population may be high and also vary by geography and type of lifestyle [2], [3], [4], [15], [16]. With this background, the purpose of this study was to assess the prevalence of diabetes mellitus and glucose intolerance and to apply multilevel regression analysis to simultaneously examine the inter-relationship between both individual and contextual socioeconomic status (SES) and diabetes mellitus and glucose intolerance in the adult population in rural southwest China.

## Methods and Subjects

### Sampling Frame

The study was cross-sectional questionnaire survey conducted in rural areas of Yunnan Province. A multistage stratified random sampling method was employed to select a representative sample. In stage one, 2 counties, one economically advantaged and the other economically disadvantaged, were selected from among all counties in Yunnan province. In stage two, to guarantee the representativeness of each sample, all current townships of the selected counties were included in the study, involving a total of 12 townships. In stage three, within each township, 2 villages were randomly selected with a selection probability proportional to the population size (population aged over 25 years). Altogether there were 24 study villages; 220 individuals were randomly recruited from each of those villages.

### Target Population

Non-institutionalized adults aged 25 years and over residing in private dwellings in each of the 12 townships were included in the survey if they had resided permanently at the address for a minimum of 6 months prior to the survey. Persons with physical or intellectual disabilities that precluded participation in the study were not included.

### Data source

The survey activities occurred over a 2-month period between May and June 2011. Approximately 1 month was allocated to the collection of contextual (township) characteristics of villages with regard to population size, adult literacy rate, proportion of minorities and rural average income (Yuan) based on the 2009 census in China in the local statistics office. The survey activities were divided into two phases - the household interview and the biomedical examination. Individual characteristics, yearly household income and known or suspected diabetes risk factor parameters were obtained from a cross-sectional community survey. Before being interviewed each participant was given a full explanation of the research purpose, and was then given the option to sign an informed consent form. If the respondent agreed to participate in the study, he or she was surveyed by trained interviewers using a pre-tested structured questionnaire. The questionnaire covered social and demographic characteristics, tobacco consumption, alcohol intake, education level, ethnicity and yearly household income. Weight, height and BMI were measured according to standard methods [17], [18]. Body mass index (BMI) was calculated as the weight (kg) divided by the square of the height (m). Three consecutive blood pressure readings, separated by at least 15 minutes, were taken from the right arm of seated subjects, and the average of the three readings was used in subsequent data analyses.

### Biomedical Examination

The biomedical examination was conducted at a local test site in each sampled area. Subjects arrived at the local test site early in the morning after an overnight fast (8–16 h). Following the initial collection of the fasting blood sample, an oral glucose tolerance test (OGTT) was performed on all participants, except those on insulin or oral hypoglycemic drugs or those who were pregnant. Plasma glucose levels were measured using the glucose oxidase method. Serum true insulin was assayed used a bio-antibody technique (Linco, St Louis, MO, USA). The criteria for diabetes mellitus issued by World Health Organization (WHO) in 1999 (fasting plasma glucose[FPG]≥7.0 mmol/l and/or 2-h postprandial plasma glucose [2hPG] ≥11.1 mmol/l)) were used. Glucose intolerance is only defined as IFG (impaired fasting glucose, FPG≥5.6 mmol/l and <6.9 mmol/l) and/or IGT (impaired glucose tolerance, 2hPG≥7.8 mmol/l and <11.1 mmol/l). For pregnant women, diabetes mellitus and glucose intolerance was identified by self-reported physician diagnosis.

### Definitions

Cigarette smoking was defined as having smoked at least 100 cigarettes in one’s lifetime. Alcohol drinking was defined as the consumption of at least 30 g of alcohol per week for 1 year or more. The criteria for hypertension issued by WHO in 1999 (systolic blood pressure of 140 mmHg and/or diastolic blood pressure of 90 mmHg) were used.

### Outcome and Independent Variables

The outcome variables included binary measures of diabetes and glucose intolerance. Independent variables included both individual and township variables. Individual characteristics included age, gender, education, yearly household income, ethnicity, BMI, smoking, drinking and hypertension. The township variables (contextual variables) were mean yearly income, percent primary (grades 1–6) education or higher, population size and percentage of ethnic minorities. Individual sex, ethnicity and education are categorical variables, and the others are continuous.

### Ethical Approval

The study protocol was approved by the Ethics Committee of Faculty of Medicine, Kunming Medical University, before carrying out the research. Written consent was obtained from all participants prior to data collection.

### Statistical Methods

All analyses were performed using R for Windows. Continuous variables were described by the mean and standard deviation. For qualitative data, group comparisons were performed using the Chi-square test or Fisher's exact test as appropriate. Age-standardized estimates of prevalence were calculated by the direct method, where the 2009 census of the Chinese adult population aged ≥25 years was taken as the standard. The data were further analyzed by means of a multilevel logistic regression model, whose estimation was performed by restricted maximum likelihood, with individual characteristics at the first level and contextual socioeconomic status at the second. The township characteristics are random effects. Interactions with the township characteristics were treated as random effects. The individual factors are fixed effects. The results are shown as odds ratios (OR) with a 95%CI. A P value <0.05 was considered significant. The fit of the model was judged from an overdispersion parameter which ideally, should be approximately 1. The fitted models met this demand. There were no significant interactions between township characteristics in the model, although all possible interactions were tested.

Multilevel (or hierarchical) regressions were carried out to take into account the hierarchical nature of the collected data (level 1: individuals; level 2: township level). Using this type of modeling allowed testing for association between the occurrence of diabetes and glucose intolerance and person-level variables (level 1) and, independent of the latter, the characteristics of the townships (level 2). In other words, we fitted two models. First, we included only individual variables in the model (model 1) to assess the extent to which the occurrence of diabetes and glucose intolerance could be explained by individual characteristics, and then added in the township variables (model 2) (in addition to the variables already included in model 1) to investigate whether the effects of individual characteristics were conditioned by their context.

## Results

All the eligible subjects in the study areas (≥25 years, n = 5280) were enlisted. More than 90.9% (n = 4801; men 44.8%, women 55.2%) responded. Characteristics of the survey population are shown in Table 1. The mean age of participants was 51.1 years old with a range of 25–86 years and about three-fourths were (76.7%) under 65 years. The percentage of ethnic minorities was 47.9%, and the adult illiteracy rate was 27.1%. Women had a higher illiteracy rate than men (32.8% versus 24.3%). Male participants had higher height, weight and yearly household income than female participants (P<0.01), whereas female participants had higher BMI (P<0.05). Smoking and alcohol consumption were almost entirely confined to men.

**Table 1:** General characteristics of the survey population.

Characteristic	Males (n = 2152)	Females (n = 2649)	All (N = 4801)
Height (cm)*	162.32±7.16	152.50±6.74	156.90±8.48
Weight (kg)*	60.20±9.23	53.57±9.01	56.54±9.68
BMI (kg/m2)^**^	22.83±3.19	23.00±3.40	22.93±3.31
Yearly household income (in Yuan)*	5211±23	4821±19	5016±22
Age (in years) N (percent)			
25-44	802 (37.3)	944 (35.6)	1746 (36.4)
45-54	405 (18.8)	550 (20.8)	955 (19.9)
55-64	443 (20.6)	546 (20.6)	989 (20.6)
≥65	502 (23.3)	609 (23.0)	1111 (23.1)
Ethnicity N (percent)			
Han	1134 (52.6)	1368 (51.6)	4302 (52.1)
minorities	1018 (47.1)	1281 (48.4)	499 (47.9)
Education			
Illiterate^**^	533 (24.3)	869 (32.8)	1302 (27.1)
≥Primary (grades 1–6)	1420 (66.0)	897 (33.9)	2317 (48.3)
Current smokerN (percent)	1479 (68.7)	13 (0.5)	1492 (31.1)
Current drinker N (percent)	929 (43.2)	75 (2.8)	1004 (20.9)
Hypertensive N (percent)**	577 (26.8)	789 (29.8)	1366 (28.4)

* p<0.01.

* * p<0.05.

Age-and gender-standardized prevalence of diabetes mellitus and glucose intolerance in participants is shown in Table 2. Overall standardized prevalence of diabetes mellitus and glucose intolerance were 7.1% (previously undiagnosed, 3.6%) and 8.8%, respectively. Prevalence of diabetes mellitus was 7.8% in women and 6.5% in men (P<0.0001), respectively. Glucose intolerance was also more common among women than men (P<0.0001). The prevalence of diabetes mellitus and glucose intolerance increased significantly with increasing age (P<0.001).

**Table 2:** Age-and gender-standardized prevalence of diabetes mellitus and glucose intolerance among participants.

Age	Diabetes Mellitus			Glucose Intolerance		
	Males (%)	Females (%)	All (%)	Males (%)	Females (%)	All (%)
25-44	3.2	3.8	3.7	5.4	5.9	6.2
45-54	5.9	6.3	6.4	8.9	9.9	9.5
55-64	6.4	8.3	7.7	8.2	9.8	9.1
≥65	11.4	13.3	12.5	11.2	13.0	12.3
All	6.5	7.8	7.1	8.0	9.5	8.8

Table 3 summarizes the contextual variables. Variations in adult mean yearly income and percentage of minority ethnic were high.

**Table 3:** Distribution of socioeconomic status among 12 villages.

Variables	Max	P75	P50	P25	Min
Mean yearly income	7853	5670	4819	4125	1253
≥Primary (grades 1–6) education (percent)	78.3	66.7	50.9	46.6	39.8
Population size (x1000) size	8011	3571	2509	1831	1254
Ethnic minorities (percentage)	100	56.7	48.3	26.6	9.8

Table 4 shows the results of the multilevel models for prevalence of diabetes mellitus and glucose intolerance. For the outcome variable being diabetes, the null multilevel model indicated an intercept variation that was significantly different from zero (random-effects variance  = 0.019; P<0.0002) (results not shown). When only individual variables (Model 1) were introduced into the null multilevel model, the random-effect variance was down to 0.012, but it remained significantly different from null multilevel model (P<0.001). When individual variables and township variables (Model 2) were introduced into the null multilevel model, the random-effect variance decreased to 0.004, but it also remained significantly different from null multilevel model (P<0.001). Models 1 and 2 explained respectively 63% and 70% of the variance in the log odds of diabetes. The fit of the models for glucose intolerance is similar to that of the diabetes models. When individual variables and township variables introduced into the null multilevel model, 68% and 74% of the variance in the log odds of glucose intolerance across township was explained by individual-level variables and both individual-level and township-levels variables respectively.

**Table 4:** Multilevel logistic regression analysis of diabetes and glucose intolerance in a community-based, cross-sectional survey of rural adults in Yunnan Province.

Predictors	Diabetes			Glucose intolerance			Diabetes			Glucose intolerance		
	OR	95%CI	P	OR	95%CI	P	OR	95%CI	P	OR	95%CI	P
Individual variables												
Age	1.18	(1.17,1.19)	0.006	1.15	(1.11, 1.19)	0.003	1.03	(1.02,1.04)	0.034	1.04	(1.03, 1.05)	0.042
Gender (reference: male)	1.95	(1.72, 1.18)	P<0.001	1.59	(1.25, 1.93)	P<0.001	2.05	(1.67, 2.43)	P<0.001	1.88	(1.56, 2.30)	P<0.001
Education level (reference: illiterate)	0.85	(0.80, 0.90)	0.023	0.75	(0.81, 1.49)	0.251	0.95	(0.91, 0.99)	0.027	0.88	(0.97, 1.54)	0.364
yearly household income (x 1000 Yuan)	0.83	(0.75, 0.91)	0.008	1.00	(1.00, 1.01)	0.160	0.89	(0.83, 0.97)	0.011	0.53	(0.41, 0.65)	P<0.001
Ethnicity (reference: han)	1.06	(1.03, 1.09)	0.035	1.47	(1.21, 1.73)	0.001	1.18	(0.86, 1.59)	0.523	1.47	(0.70, 1.14)	0.123
BMI	1.31	(1.09, 1.54)	P<0.001	1.08	(1.03, 1.13)	0.017	1.50	(1.21, 1.84)	0.021	1.16	(1.11, 1.21)	0.025
Current smoking (reference: nonsmoking)	0.86	(0.81, 0.91)	0.028	1.32	(0.65, 1.80)	0.331	0.86	(0.81, 0.91)	0.008	1.45	(0.87, 2.02)	0.338
Current drinking (reference: nondrinking)	1.00	(1.00, 1.01)	0.639	1.03	(1.01, 1.05)	0.042	1.13	(1.10, 1.16)	0.001	1.08	(1.02, 1.14)	0.016
Hypertensive (reference: non hypertensive)	0.93	(0.82, 1.04)	0.091	0.90	(0.85, 0.95)	0.029	0.9	(0.81, 1.01)	0.191	0.98	(0.93, 1.03)	0.075
Contextual variables												
Mean yearly income							0.91	(0.84, 0.98)	0.0232	0.89	(0.85, 0.93)	0.010
Percent primary (grades1–6) education or higher							0.97	(0.95, 0.99)	0.038	1.03	(0.78,1.22)	0.506
Population size (x1000)							1.00	(0.99, 1.00)	0.093	1.00	(0.99, 1.00)	0.218
Percentage of ethnic minorities							1.11	(1.07, 1.15)	0.014	0.99	(0.98, 1.00)	0.469
Explained variance (%)	63			68			79			74		

OR =  odds ratio.

CI: confidence interval.

For individual demographic variables, females had a higher likelihood of diabetes mellitus and glucose intolerance than males (2.05 fold and 1.88 fold, respectively). The occurrence of diabetes mellitus and glucose intolerance increased with age and BMI, but decreased with yearly household income. Subjects with higher education level were less likely to suffer from diabetes mellitus compared to those with lower education level. Further, percent primary (grades 1–6) education, as one of the township-level variables, also showed a significant inverse association with the prevalence of diabetes mellitus. Current drinkers were more likely to suffer from diabetes mellitus and glucose intolerance than non-current drinkers. In contrast, smoking individuals were significantly less likely to suffer from diabetes mellitus.

Township-level variables in the multilevel model showed that the mean yearly income was significant inversely associated with the prevalence of diabetes mellitus and glucose intolerance. In other words, residents of villages with higher mean yearly income had a lower likelihood of diabetes mellitus and glucose intolerance. Those in communities with higher literacy rate had decreased probability of diabetes mellitus. Although percentage of ethnic minorities was not associated with prevalence of glucose intolerance, individuals in areas with high percentage of ethnic minorities had an increased probability of diabetes mellitus.

## Discussion

China has experienced a high-speed socioeconomic development after reforming and opening up in 1980s, resulting in rapid modernization and urbanization. Simultaneously, a three-fold increase in prevalence of diabetes mellitus, from 1% in 1980 to 3.2% in 1996, was found in Chinese adults [19], [20]. A recent Chinese study using a representative sample of the whole national population shows that the total prevalence of diabetes mellitus was 9.7%, and among rural residents the prevalence of diabetes mellitus was 8.2%, lower than among urban residents [21]. In our study, there also was a high percentage prevalence of diabetes mellitus (7.1%) in contrast to 5.6% reported in a previous study [22]. There were many reasons for the high prevalence of diabetes mellitus, including individual variables and neighborhood environment. Some studies have suggested that local geographical variations and socioeconomic position are related to diabetes mellitus prevalence [11], [12].

This study examined the influence of contextual variables such as mean yearly income, percent primary education, population size (x1000) and percentage of ethnic minorities on diabetes mellitus and glucose intolerance in a regional sample of women and men in rural southwest China using multilevel models. Consistent with our expectations, we found that persons with lower socioeconomic position (SEP) consistently have higher likelihood of diabetes mellitus compared to those with higher SEP when adjusted for age and sex. Some studies also reported that diabetes mellitus was associated with low socioeconomic position [23], [24]. For example, educational level was a good indicator of socioeconomic status, and a higher educational level has been associated with lower levels of diabetes risk factors, such as obesity, physical inactivity and an unhealthful diet [25]. Our study also showed a significant inverse association between individuals’ and townships’ education levels and diabetes. A National Health Interview Survey in the U.S showed that educational attainment was inversely associated with the prevalence of diabetes mellitus in US adults [15]. Ko et al. concluded that a lower socioeconomic class categorized by educational level was an additional risk factor for diabetes mellitus in Hong Kong Chinese [6]. The inverse association between township education level (percent of primary grades) and diabetes mellitus suggests that the educated rural communities rather than the uneducated ones are still being targeted for future intervention programs.

Further, as another indicator of socioeconomic status, yearly household income was inversely related to diabetes mellitus and glucose intolerance in our study. In other words, participants have higher likelihood of diabetes mellitus and glucose intolerance in low-income groups compared to higher-income groups. Increased diabetes mellitus and glucose intolerance prevalence in low-income groups has also been documented in other population-based studies [26], [27], [28]. However, converse findings have been reported in China 1994 [29]. Further studies are required in order to shed light on the true effects of income on diabetes.

In our studies the frequency of diabetes and glucose intolerance was comparable, and both were moderately high by international standards [30]. Compared to other surveys that used WHO diagnostic criteria in other countries or regions in East Asia, the prevalence of diabetesin rural adults of southwest China is lower than that in Taiwan [31], in Singapore [32], Hong Kong [33]; similar to that in the Turkic population [34]; higher than the data of 1994 in China [29]. In addition, the prevalence of diabetes mellitus and of glucose intolerance, which is an important risk factor in the development of clinical diabetes, was significantly lower than that in the data of 2008 in in rural China [21]. Other important points of the study may be that the proportion of undiagnosed diabetes mellitus was extremely high; more than one out of every three people with diabetes mellitus are undiagnosed. Similar findings have been reported in other Asian populations [35], [36], [37]. This result indicates a lack of public awareness and shortage of medical facilities in rural areas, and it is urgent to establish a national education program in rural China to promote regular community and clinic-based diabetes screening for the early detection of diabetes mellitus and glucose tolerance.

Like previous studies [38], [39], it appears that in this study, diabetes mellitus and glucose intolerance were more common in women than in men. Further, in all age categories the prevalence of diabetes mellitus and glucose intolerance was higher in females compared to males and glucose intolerance was more common than diabetes mellitus in rural areas of southwest China. Over more than 30 years, the reform and opening up has promoted an accelerated urbanization in China, resulting in amazing changes in lifestyle. Rural women are no longer exceptionally hard-worked in the fields as in previous times and their physical activity is now restricted to housework, and women have no tradition for sporting activities. So physical inactivity and an unhealthful diet may contribute to the higher frequency of obesity and glucose intolerance among rural women.

In our study, BMI was positively associated with the frequency of diabetes mellitus and glucose intolerance. The finding that these factors were important contributors to both diabetes mellitus and glucose intolerance has been demonstrated in many previous studies [40], [41], which indicated that overweight and obesity increased the risk of diabetes mellitus and glucose intolerance. The mechanisms by which obesity and centralized fat distribution are associated with increased diabetes risk are poorly understood, but a finding in 2000 suggested that excess body fat, especially visceral obesity, increased insulin resistance by releasing free fatty acids and cytokines that interfere with insulin action, along with other deleterious effects that could lead to diabetes mellitus [42].

Though several previous studies [43], [44], [45] showed an inverse association between light-to-moderate alcohol consumption and the development of diabetes mellitus, our study showed a positive association between alcohol intake and risk of diabetes mellitus. A similar phenomenon has been demonstrated in other studies [46], [47]. The reason for this dichotomy may be excessive drinking behavior in ethnic minority population, and some studies [48], [49] have demonstrated that ethnic minorities have more frequent alcohol intake than Han majority. According to the present study, smoking was inversely related to the risk of diabetes but not to that of glucose intolerance; this result was also in line with previous results [8], [34].

Neither individual nor township ethnic minority variables influenced glucose intolerance in this study, whereas percentage of ethnic minorities was significant associated with frequency of diabetes. Similar results also appeared in other studies [16], [50]. The ethnic differences in diabetes prevalence indicates that other features such as genetic susceptibility, lifestyles or environmental factors may be responsible for the higher frequency of diabetes among ethnic minorities. The results suggests that ethnicity is an important consideration in the management of diabetes, but further research was required to find out if this is true.

These populations should have increased priority for health education and regular diabetes screening. Our findings further emphasize the complex relationships between diabetes and glucose intolerance contextual socioeconomic determinants.

## Limitations

This analysis was subject to limitations. First, as this was a cross-sectional survey, causal effects could not be established with certainty. Second, our findings may be subject to recall bias. Last, as our sampling was limited to only two counties in Yunnan province, we could not claim that our findings were representative for all counties in China, but the results do provide useful insights and suggest possible interventions to eliminate diabetes risk factors for people living in rural areas.

## Conclusion

This survey has shown that the prevalence of diabetes mellitus and glucose intolerance was high in the rural southwest China population. The higher prevalence of glucose intolerance was probably an indicator of a further rise in prevalence of diabetes in the years to come. The results of this study suggest that the study region should emphasize further control of glucose intolerance, and future strategies for diabetes prevention should address the contextual in addition to the person-centered interventions.
